# Cognitive impairment and medication adherence post-stroke: A five-year follow-up of the ASPIRE-S cohort

**DOI:** 10.1371/journal.pone.0223997

**Published:** 2019-10-17

**Authors:** Daniela Rohde, Eva Gaynor, Margaret Large, Lisa Mellon, Kathleen Bennett, David J. Williams, Linda Brewer, Patricia Hall, Elizabeth Callaly, Eamon Dolan, Anne Hickey

**Affiliations:** 1 Division of Population Health Sciences, Royal College of Surgeons in Ireland, Dublin, Ireland; 2 Department of Medicine, Royal College of Surgeons in Ireland, Dublin, Ireland; 3 Clinical Research Centre, Royal College of Surgeons in Ireland, Beaumont Hospital, Dublin, Ireland; 4 Geriatric and Stroke Medicine, Royal College of Surgeons in Ireland and Beaumont Hospital, Dublin, Ireland; 5 Geriatric Medicine, Mater Misericordiae University Hospital, Dublin, Ireland; 6 Geriatric Medicine, Connolly Hospital, Dublin, Ireland; University of Central Florida, UNITED STATES

## Abstract

**Background:**

Control of vascular risk factors is essential for secondary stroke prevention. However, adherence to secondary prevention medications is often suboptimal, and may be affected by cognitive impairment. Few studies to date have examined associations between cognitive impairment and medication adherence post-stroke, and none have considered whether adherence to secondary prevention medications might affect subsequent cognitive function. The aim of this study was to explore prospective associations between cognitive impairment and medication non-adherence post-stroke.

**Methods:**

A five-year follow-up of 108 stroke survivors from the Action on Secondary Prevention Interventions and Rehabilitation in Stroke (ASPIRE-S) prospective observational cohort study. Cognitive function was assessed using the Montreal Cognitive Assessment at 6 months, and a neuropsychological test battery at 5 years. Adherence to antihypertensive, antithrombotic and lipid-lowering medications was assessed using prescription refill data.

**Results:**

The prevalence of cognitive impairment at five years was 35.6%. The prevalence of non-adherence ranged from 15.1% for lipid-lowering agents to 30.2% for antithrombotics. There were no statistically significant associations between medication non-adherence in the first year post-stroke and cognitive impairment at 5 years, nor between cognitive impairment at 6 months and non-adherence at 5 years. Stroke survivors with cognitive impairment were significantly more likely to report receiving help with taking medications [OR (95% CI): 4.84 (1.17, 20.07)].

**Conclusions:**

This is the first study to explore the potential impact of non-adherence to secondary prevention medications on cognitive impairment in stroke survivors. Findings highlight the role of family members and caregivers in assisting stroke survivors with medication administration, particularly in the context of deficits in cognitive function. Involving family members and caregivers may be a legitimate and cost-effective strategy to improve medication adherence in stroke survivors.

## Introduction

Cognitive impairment is common post-stroke and can increase levels of disability and dependency, leading to a greater burden on caregivers and the healthcare system [1–4]. Approximately 10% of stroke survivors experience dementia [5], with an estimated 38% experiencing cognitive impairment that does not meet the criteria for dementia within 12 months post-stroke [6]. Cognitive impairment adversely impacts independence in activities of daily living and may affect the ability to adhere to medications to control secondary risk factors [7–9]. Vascular risk factors, including hypertension, dyslipidemia, and atrial fibrillation, are associated with an increased risk of cognitive impairment [8, 10, 11], while adequate risk factor control could significantly reduce this risk [12]. However, adherence to medications to control these risk factors is frequently sub-optimal [13].

Few studies to date have examined associations between cognitive impairment and medication adherence post-stroke, with a recent systematic review finding no association between cognitive impairment and adherence when all studies were pooled, although heterogeneity was significant and overall evidence quality was poor [14]. Given the variety of assessments of both medication adherence and cognitive impairment, it is difficult to compare findings across studies. Further, all studies to date have examined cognitive impairment as a predictor of medication non-adherence; whether adherence to secondary prevention medications might affect subsequent cognitive impairment remains unexplored [14]. Medication adherence consists of three distinct phases: initiation, implementation or persistence, and discontinuation or non-persistence [15], but which phase of adherence is being assessed is often not adequately reported. This study focused on the implementation phase of adherence, and assessed adherence using both self-report and prescription refill data in an attempt to better capture medication (non)adherence [16]. The main aims of this study were: 1) to explore the prospective association between cognitive impairment at six months and adherence to secondary prevention medications at five years post-stroke, and 2) to explore the prospective association between medication non-adherence at 12 months and cognitive impairment at five years post-stroke.

## Materials and methods

### Study design

This study involved a five-year follow-up of the Action on Secondary Prevention Interventions and Rehabilitation in Stroke (ASPIRE-S) observational cohort study, which recruited acute ischemic stroke survivors in hospital and followed them up in the community six months later [17, 18]. The design and methods of this five-year follow-up have been described previously [19, 20].

### Participants

**Fig 1:**
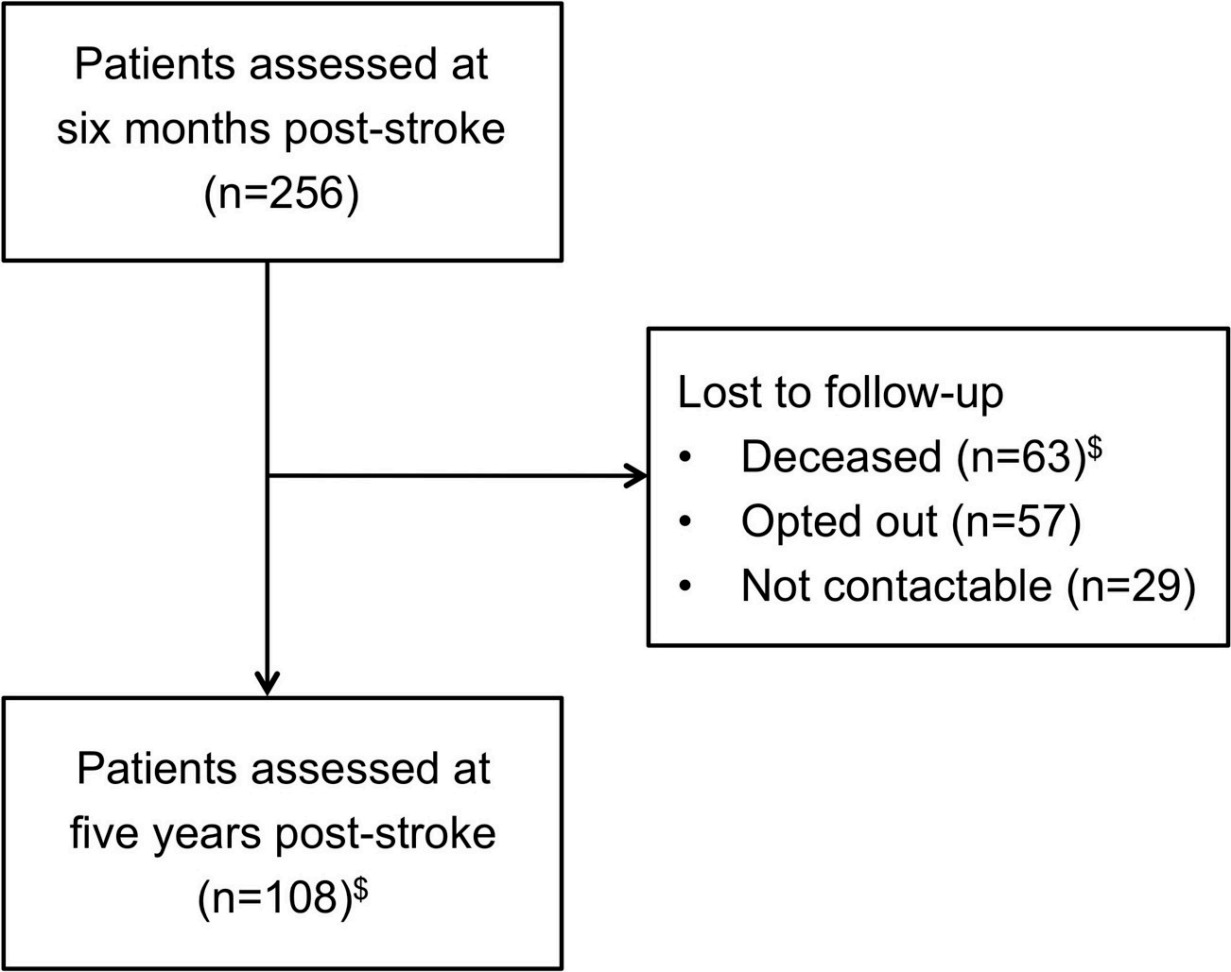
Flowchart of ASPIRE-S stroke survivors. ^$^One participant was followed up at five years, but subsequently died before the end of the study period.

All stroke survivors who were alive at five years were eligible to participate. Of 256 participants assessed at six months post-stroke, 63 (24.6%) died within five years, 57 (22.3%) opted out and 29 (11.3%) were not contactable, leaving 108 stroke survivors assessed at five years post-stroke (2016–2017) (Fig 1). The mean follow-up was 5.1 years (SD 0.4) from the index stroke event.

### Data collection and analysis

Data were collected using a combination of face-to-face assessments either in participants’ own homes or one of the participating hospitals, and self-completion questionnaires.

#### Cognitive assessments

Global cognitive function was assessed at six months and five years post-stroke using the Montreal Cognitive Assessment (MoCA), a 30-point screening tool that assesses several cognitive domains [21]. Concerns have been raised over the lack of specificity of the original cut-off (<26), with some authors recommending more conservative cut-offs (e.g., <24) [22–24].

In addition to the MoCA, cognitive impairment at five years was assessed using the National Institute of Neurological Disorders and Stroke (NINDS) 30-minute test battery [25], including Digit Symbol Coding (DSC) from the Wechsler Adult Intelligence Scale [26], Verbal and Letter Fluency from the Delis-Kaplan Executive Function System [27], Hopkins Verbal Learning Test (HVLT) [28], and Trail Making Test parts A and B (TMT-A, TMT-B) [29]. Raw test scores for each task were transformed into z-scores according to published age-, and where available, education-adjusted normative means and standard deviations [26, 27, 30, 31]. For each cognitive assessment, a composite z-score was calculated using equal weights; for example for TMT: 0.5*TMTA+ 0.5*TMTB [32]. A composite executive function z-score was then created using equal weights consisting of the composite TMT A/B, verbal/letter fluency and DSC. For patients who did not have all assessments available, the composite used z-scores from the completed assessments, e.g., category and letter fluency for patients who were visually impaired or unable to use a pen to complete TMT or DSC. Memory was assessed using HVLT total recall score. Patients were classified as cognitively impaired if they had evidence of impairment in at least one of the two domains according to scores 1.5SD below norms [31, 33, 34]. 102 of 108 stroke survivors followed up at five years completed the NINDS neuropsychological test battery; 6 participants were unable or unwilling to complete the assessments due to fatigue or severe cognitive, language, or functional impairments.

#### Medication adherence

Self-reported adherence at six months and five years post-stroke was assessed using the Medication Adherence Rating Scale (MARS-5) [35]. The MARS-5 consists of 5 items relating to medication-taking behaviour, including forgetting to take medications, or altering, skipping or missing doses. Respondents rate their own use of medications according to each item on a scale from 1 (very often) to 5 (never). Responses are summed to provide a total score, with higher scores indicating better self-reported adherence. Due to a ceiling effect, we used a cut-off of <25 to identify non-adherence [36, 37].

For stroke survivors with available data, medication adherence in the year following stroke was assessed using prescription refills. Data was extracted from the Irish Health Service Executive Primary Care Reimbursement Services (HSE-PCRS) pharmacy claims database. This database contains all monthly-dispensed medications for each individual eligible for the General Medical Services (GMS) reimbursement scheme, which includes access to free healthcare and a small co-payment for medicines. Further details of the HSE-PCRS are available elsewhere [19, 38]. We considered medications commonly used for secondary prevention of ischemic stroke, categorized according to three therapeutic groups: antihypertensives, antithrombotics (anticoagulant/antiplatelet), and lipid lowering medications [39, 40]. We assessed medication adherence according to the proportion of days covered (PDC) for the 12 months following the stroke event: the total number of days of medications supplied within each therapeutic class, divided by 365 [16]. A cut-off of <80% is commonly used to identify non-adherence in populations with cardiovascular disease and stroke, and was applied in this study [16, 41]. Medication adherence at five years post-stroke according to prescription refills was calculated in the same way, by considering the 12 months prior to the five-year follow-up assessment. Prescription refill data was available for 53 stroke survivors at five years. Missing data was due to participants’ ineligibility for the GMS scheme, or failure to provide the necessary information to allow linkage between the HSE-PCRS and the ASPIRE-S databases.

#### Covariates

Stroke severity was assessed using the Scandinavian Stroke Scale [42]. TOAST (Trial of Org 10172 in Acute Stroke Treatment) [43] and Bamford [44] classifications of the index stroke event were collected as part of the original ASPIRE-S study. Functional disability at 6 months post-stroke was assessed using the modified Rankin Scale (mRS) [45] with a cut-off of ≥3 to identify stroke survivors with moderate to severe functional disability [46]. Depressive symptoms at five years were assessed using the Center for Epidemiologic Studies Depression Scale (CES-D), with a cut-off of ≥16 to identify depressive symptoms [47]. The Frenchay Aphasia Screening Test (short form) (FAST) [48] was used at both six months and five years post-stroke to screen for communication difficulties that may affect performance on cognitive assessments. Clinical measures collected at both six month and five years post-stroke included blood pressure, lipid profiles, fasting glucose levels, weight, history of stroke/TIA, history of heart disease, history of carotid stenosis and presence of atrial fibrillation. Vascular risk factors were classified according to European secondary prevention targets [49]. Given the number of vascular risk factors, the Essen Stroke Risk Score (ESRS), a 10-point scale designed to predict 1-year risk of recurrent stroke and cardiovascular events [50–52], was calculated and included in multivariate analyses. Medication self-administration was ascertained by a single question asking stroke survivors whether they received help with medication taking.

#### Ethical considerations

All procedures performed in this study were in accordance with ethical standards of the institutional research committees and with the 1964 Helsinki declaration and its later amendments. Written informed consent was obtained from all individual participants included in the study. This study followed the UK Medical Research Council (MRC) guidelines for research involving adults who cannot consent [53], and the Irish National Consent Policy [54]. According to both guidelines, every potential participant should be presumed to have the capacity to make decisions about participation in research, unless there is sufficient reason to question this presumption. The policies suggest that the possibility of incapacity and the need to formally assess capacity should be considered only if, having been given all appropriate help and support, an individual is unable to communicate a clear and consistent choice, or is obviously unable to understand and use the information and choices provided. If the person was deemed, either by their general practitioner, their family member or carer, or the member of the research team conducting the study, to lack the capacity to consent, agreement to include them in the study was sought from a relative or carer [53]. If the relative or carer advised that the person in question would not want to take part in the study, that person was not recruited. In addition, participants who indicated any unwillingness or objection to participation in the study were not recruited [53]. This procedure was approved by the research ethics committees at Beaumont Hospital (REC number: 16/26), Mater Misericordiae University Hospital (REC number: 1/378/1855), Connolly Hospital Blanchardstown (REC number: 28/11/2016), and the Royal College of Surgeons in Ireland (REC number: 1355).

### Data analysis

Descriptive statistics are presented using frequencies and percentages. Univariate associations between demographic and clinical variables and cognitive impairment/medication adherence were explored using chi-square and t-tests as appropriate. Associations between cognitive impairment and medication adherence were explored using logistic regression models, adjusted for age, sex, and stroke severity and Essen stroke risk score. Odds ratios (OR) and 95% confidence intervals (CI) are reported. To maximize available data, pairwise deletion of missing data was used. Sensitivity analyses were conducted excluding stroke survivors with possible aphasia. Agreement between medication non-adherence according to prescription refills and self-report was assessed using the kappa statistic. We evaluated the predictive values of MoCA cut-offs (<26 and <24) for cognitive impairment according to the NINDS battery using a receiver operating characteristic (ROC) curve analysis [55]. Data were analyzed using Stata version 13.0 [56].

## Results

Demographic, cognitive and medication adherence profiles of stroke survivors at five years post-stroke are presented in Table 1. Thirty-six stroke survivors (35.6%) had evidence of cognitive impairment at five years according to the NINDS battery. When survivors with probable aphasia were excluded, 28.3% had evidence of cognitive impairment. For cognitive and medication adherence profiles of stroke survivors at five years post-stroke according to sex, please see S1 Table in the Supporting Information. At five years post-stroke, MoCA scores <26 had a sensitivity of 94.4% and specificity of 56.9% for cognitive impairment according to the more detailed NINDS test battery in our sample (AUROC 0.757), while the cut-off of <24 substantially increased specificity (78.1%), with a sensitivity of 88.2% (AUROC 0.832).

**Table 1:** Demographic, cognitive, and medication adherence profiles of stroke survivors at five years post-stroke.

**Demographics**		**N (%)**
Sex (n = 108)	Male	73 (67.6)
	Female	35 (32.4)
Marital status (n = 101)	Married/cohabiting	65 (64.4)
	Single/widowed/divorced	36 (35.6)
Living arrangements (n = 100)	Living alone	26 (26.0)
	Living with others	71 (71.0)
	Nursing home resident	3 (3.0)
Occupational status (n = 94)	Working full-time or part-time	18 (19.2)
	Not working	76 (80.9)
Education (n = 98)	Primary school	31 (31.6)
	Secondary school	42 (42.9)
	Third level	25 (25.5)
**Cognitive impairment**		**Impaired** **N (%)**
Memory (n = 100)	HVLT total recall	23 (23.0)
Executive function (n = 102)	Semantic fluency	22 (21.6)
	Letter fluency	28 (27.5)
	Digit symbol coding (n = 96)	31 (32.3)
	Trail Making Test–A (n = 93)	14 (15.1)
	Trail Making Test–B (n = 92)	23 (25.0)
	Composite executive function	24 (23.8)
Impaired in both NINDS domains (n = 102)		11 (10.8)
Impaired in at least one NINDS domain (n = 102)		36 (35.6)
MoCA <24 (n = 101)		46 (45.5)
**Medication adherence**		**Non-adherent** **N (%)**
Prescription refills (PDC<80%) (n = 53)	Lipid modifiers	8 (15.1%)
	Antithrombotics	16 (30.2%)
	Antihypertensives	12 (22.6%)
Self-report (MARS) (n = 95)		51 (53.7%)

NINDS: National Institute of Neurological Disorders and Stroke. MoCA: Montreal Cognitive Assessment. MARS: Medication Adherence Report Scale.

Table 2 displays the demographic and clinical profile of ASPIRE-S stroke survivors by cognitive status according to the NINDS 30-minute test battery at five years post-stroke. Older age, impaired fasting glucose, history of carotid stenosis, previous or recurrent stroke/TIA and higher Essen Stroke Risk Score at five years were associated with significantly increased likelihood of cognitive impairment at five years post-stroke. For demographic and clinical profiles of ASPIRE-S stroke survivors by medication adherence status at five years post-stroke, please see S2 Table in the Supporting Information. Stroke survivors with evidence of cognitive impairment at five years were also significantly more likely to receive help with taking medications [aOR (95% CI): 4.84 (1.17, 20.07)] (Table 3).

**Table 2:** Demographic and clinical profile of ASPIRE-S stroke survivors at six months post-stroke by cognitive status (NINDS) at five years.

Demographics, index stroke characteristics and clinical risk factors at five years		Not impaired	Cognitive impairment	*p*
		N (%)		
**Demographics** **@ 5 years**	Age (Mean, SD)	66.2 (12.3)	73.2 (11.9)	.006**
	Male	47 (71.2)	22 (61.1)	.297
	Married (vs. not married)	45 (71.4)	17 (50.0)	.036*
**TOAST classification**	Large artery artherosclerosis	10 (15.2)	8 (22.2)	.364
	Cardioembolism	20 (30.3)	15 (41.7)	
	Small vessel occlusion	11 (16.7)	4 (11.1)	
	Other	25 (37.9)	9 (25.0)	
**Bamford classification**	Total anterior circulation stroke	4 (6.1)	2 (5.6)	.901
	Partial anterior circulation stroke	22 (33.3)	15 (41.7)	
	Posterior circulation syndrome	21 (31.8)	9 (25.0)	
	Lacunar syndrome	18 (27.3)	9 (25.0)	
	Unclassifiable	1 (1.5)	1 (2.8)	
**Stroke severity**	Moderate or severe	9 (13.6)	5 (13.9)	.972
**Disability @ 6 months**	Moderate or severe	9 (13.6)	5 (13.9)	.972
**Vascular risk factors** **@ 5 years**	Hypertension	42 (63.6)	25 (69.4)	.555
	Elevated total cholesterol	20 (30.3)	9 (25.7)	.628
	Impaired fasting glucose	8 (12.1)	10 (29.4)	.033*
	Overweight/obese	50 (76.9)	24 (70.6)	.491
	Smoker	12 (18.2)	4 (11.1)	.348
	History of alcohol abuse	14 (21.2)	10 (27.8)	.455
	Previous or recurrent stroke/TIA	12 (18.2)	15 (41.7)	.010*
	History of heart disease	20 (30.3)	14 (38.9)	.379
	History of carotid stenosis	8 (12.1)	10 (27.8)	.047*
	History of atrial fibrillation	28 (42.4)	14 (38.9)	.729
	Essen Stroke Risk Score (M, SD)	2.4 (1.3)	3.4 (1.7)	< .001***
**Depression @ 5 years**	Depressive symptoms	13 (20.6)	13 (38.2)	.062

*p < .05,

**p < .01,

***p < .001.

**Table 3:** Adjusted Odds ratios (95% CIs) for help received with taking medications at 5 years and cognitive impairment and medication adherence.

			Self-administers N (%)	Receives help N (%)	aOR (95% CI)	*p*
**Cognitive impairment**	At 5 years (NINDS)	Not impaired	54 (88.5)	7 (11.5)	4.84 (1.17, 20.07)*	.030
		Impaired	21 (61.8)	13 (38.2)		
	At 6 months (MoCA)	Not impaired	60 (92.3)	5 (7.7)	5.19 (1.21, 22.22)*	.027
		Impaired	14 (53.9)	12 (46.2)		
**Medication adherence** **at 5 years**	Lipid modifiers (refills)	Adherent	28 (70.0)	12 (30.0)	1.39 (0.15, 12.54)	.767
		Non-adherent	6 (75.0)	2 (25.0)		
	Antithrombotics (refills)	Adherent	24 (68.6)	11 (31.4)	0.57 (0.10, 3.16)	.516
		Non-adherent	10 (76.9)	3 (23.1)		
	Antihypertensives (refills)	Adherent	26 (66.7)	13 (33.3)	0.36 (0.03, 4.08)	.410
		Non-adherent	8 (88.9)	1 (11.1)		
	MARS (self-report)	Adherent	32 (72.7)	12 (27.3)	0.66 (0.18, 2.42)	.533
		Non-adherent	44 (86.3)	7 (13.7)		

*p < .05. aOR adjusted for age, sex, stroke severity

Non-adherence at five years according to prescription refills ranged from 15.1% for lipid lowering agents to 30.2% for antithrombotic medications. Poor self-reported adherence was noted in 53.7% of stroke survivors (Table 1). Agreement between self-report and prescription refills was poor: 56.3% for antithrombotic medications (kappa 0.125), 54.2% for lipid modifying medications (kappa 0.083), and 52.1% for antihypertensive medications (kappa 0.042). A higher proportion of adherent stroke survivors reported that they received help with taking medications than did non-adherent individuals, although these results were not statistically significant (Table 3).

### Medication non-adherence and cognitive impairment

There were no statistically significant associations between non-adherence to any of the three medications in the first year and cognitive impairment at five years (Table 4). The exclusion of 7 individuals with probable aphasia did not greatly change these effect estimates. Similarly, there were no statistically significant associations between cognitive impairment at six months and non-adherence to lipid modifiers or antihypertensive medications at five years (Table 5). Cognitive impairment at six months post-stroke was associated with significantly reduced likelihood of non-adherence to antithrombotic medications at five years post-stroke [OR (95% CI): 0.14 (0.02, 0.90)]; however, when 3 stroke survivors with probable aphasia were excluded, this association was no longer statistically significant [OR (95% CI): 0.16 (0.02, 1.12)]. Stroke survivors with cognitive impairment at six months were 5 times more likely to report receiving help with medication taking at five years [OR (95% CI): 5.19 (1.21, 22.22)] (Table 3).

**Table 4:** Adjusted ORs (95% CI) for medication non-adherence at 6 months and cognitive impairment at 5 years.

Non-adherence at 6 months		Cognitive impairment at 5 years aOR (95% CI)^a^							
		Including stroke survivors with probable aphasia			excluding stroke survivors with probable aphasia (n = 7)				
		NINDS	*p*	MoCA	*p*	NINDS	*p*	MoCA	*p*
Prescription refills (PDC<80%)	Lipid modifiers	3.31 (0.35, 31.46)	.298	1.67 (0.17, 16.64)	.664	3.53 (0.34, 37.14)	.293	2.99 (0.21, 42.05)	.416
	Antihypertensives	0.57 (0.07, 4.34)	.583	0.69 (0.09, 5.28)	.718	0.25 (0.02, 3.34)	.298	0.38 (0.04, 3.64)	.402
	Antithrombotics	0.93 (0.19, 4.62)	.928	1.41 (0.23, 8.79)	.713	0.99 (0.16, 6.11)	.995	1.46 (0.20, 10.78)	.713
Self-report	MARS	1.12 (0.45, 2.75)	.814	0.62 (0.57, 1.54)	.307	1.62 (0.57, 4.62)	.366	0.81 (0.30, 2.15)	.666

^a^adjusted for age, sex, stroke severity, Essen stroke risk score.

**Table 5:** Adjusted ORs (95% CI) for cognitive impairment at 6 months and medication non-adherence at 5 years.

Cognitive impairment at six months post-stroke (MoCA<24)	Non-adherence at five years post-stroke aOR (95% CI)					
	Including stroke survivors with probable aphasia			Excluding stroke survivors with probable aphasia (n = 3)		
	Prescription refills			*p*	Prescription refills	*p*
	Lipid modifiers ^a^	0.09 (0.01, 1.67)		.104	0.09 (0.01, 1.88)	.121
	Antihypertensives ^b^	0.38 (0.07, 2.25)		.289	0.48 (0.08, 3.03)	.436
	Antithrombotics ^a^	0.14 (0.02, 0.90)		.038*	0.16 (0.02, 1.12)	.064
	Self-report			*p*	Self-report	*p*
	MARS		0.78 (0.27, 2.25)	.644	0.80 (0.26, 2.48)	.702

^a^adjusted for age, sex, stroke severity, Essen stroke risk score.

^b^due to collinearity with stroke severity, this model is adjusted for age, sex, Essen stroke risk score only.

*p < .05.

## Discussion

This study found no statistically significant associations between cognitive impairment assessed at six months post-stroke and medication non-adherence at five years, which is in line with the findings from a recent meta-analysis [14]. Previous research on the association between cognitive impairment and medication adherence in stroke survivors has considered cognitive impairment as one of a range of potential predictors of poor adherence, with discordant findings [14]. This is, to our knowledge, the first study to explore the potential impact of non-adherence to secondary prevention medications on subsequent cognitive impairment in stroke survivors. There were no statistically significant associations between non-adherence to antihypertensives or lipid lowering agents in the first year post-stroke and cognitive impairment at five years. While cognitive impairment at six months post-stroke was associated with significantly reduced likelihood of non-adherence to antithrombotic medications at five years, this association was no longer statistically significant when stroke survivors with evidence of aphasia were excluded.

These effect estimates suggest that stroke survivors with cognitive impairment at six months post-stroke may be more likely to have good adherence to medications at five years, although this is speculative as results were not statistically significant and should be interpreted with caution. A larger, adequately powered study would be needed to further explore this potential finding. It is important to note that stroke survivors with evidence of cognitive impairment were significantly more likely to report receiving help with taking medications, which might explain the direction of these effects. Our findings highlight the important role of family members and caregivers in assisting stroke survivors with medication administration [57], particularly in the context of cognitive impairment [58, 59].

We found that 35.6% of stroke survivors at five years had evidence of cognitive impairment according to a neuropsychological test battery, while 45.5% were categorized as impaired according to the MoCA. Previous research has reported evidence of cognitive impairment according to the MoCA in 84% of stroke survivors at 4 years [60] and in 61% of stroke survivors at 10 years post-stroke [61]. Differences in these estimates may be explained by the use of different cut-offs; using the originally recommended cut off of <26 on the MoCA, the prevalence of cognitive impairment at five years post-stroke in our sample was 62.9%. While the MoCA was designed as a screening tool, the NINDS battery may present a more robust estimate of cognitive impairment, which is in line with the reported prevalence of cognitive impairment of 38% at 12 months post-stroke according to a recent meta-analysis [6].

Previous estimates of medication adherence have been heterogeneous, with a meta-analysis of stroke survivors reporting a pooled rate of non-adherence of 30.9% [13]. One of the difficulties in medication adherence research is the absence of a gold standard measure and the related problem of the wide range of definitions and measures used, which make comparisons between studies difficult [62]. We found poor levels of agreement between self-reported and prescription refill adherence, which is in contrast to a recent study of Iranian stroke survivors [63]. However, that study considered the MARS as a continuous variable and excluded patients with moderate to severe levels of cognitive impairment [63]. Using a cut-off of <25 to identify poor self-reported adherence in our study may have led to an overestimate of non-adherence. A cut-off of <24 would classify 23.2% of our sample as non-adherent, which although more in line with the prescription refill estimates, would not have improved levels of agreement between the two measures of adherence. As self-report measures can be affected by social desirability and recall biases, which could be especially problematic in the context of cognitive impairment [16], we used the higher cut-off. However, objective assessments of adherence are likely to be more appropriate in stroke populations, which are characterized by higher levels of cognitive impairment and complex medication regimens.

A fifth of our sample reported receiving help with medication taking. A recent UK survey reported that more than half of stroke survivors living in the community were receiving some help with taking medications, including help with collecting and filling prescriptions and opening pill boxes [57]. Our single question may not have captured all of the ways in which stroke survivors may receive assistance with taking medications, which might explain these differences. Given the prevalence of non-adherence to secondary prevention medications after stroke, interventions to improve adherence have the potential to significantly improve stroke outcomes. However, a number of systematic reviews have noted that interventions to improve adherence in stroke survivors have generally lacked effectiveness [40, 62]. An important point to consider when developing and testing interventions is the extent to which family members or caregivers assist with medication taking, as a sizeable proportion of stroke survivors receive some assistance, particularly where there is cognitive impairment. Caregiver-related factors, including caregiver self-efficacy, cognitive functioning, and health knowledge, may also be associated with medication adherence [64]. Involving both stroke survivors and family members or caregivers could therefore improve the effectiveness of interventions to increase medication adherence.

### Strengths and limitations

This study has a number of strengths, including the length of follow-up, the use of prescription refill data as an objective measure of adherence and a neuropsychological test battery as a more robust assessment of cognitive impairment. Prescription refills present an efficient and objective method for calculating medication adherence estimates that are comparable to electronic measures, and may be more reliable than patient self-report [38, 41]. The majority of previous studies reporting on the association between cognitive impairment and medication adherence in stroke have not included information on whether medications were self-administered, and our results highlight the importance of considering help received with medication taking, particularly in the context of cognitive impairment.

There were a number of limitations. We previously reported that stroke survivors with cognitive impairment at six months post-stroke were more likely to have died within the follow-up period [20]. Additionally, stroke survivors who were lost to the 5-year ASPIRE-S follow-up were more likely to be older, female, and to have evidence of cognitive impairment and moderate to severe disability at 6 months post-stroke [9], suggesting that the prevalence of cognitive impairment reported here is likely to be underestimated. This reflects longitudinal stroke studies internationally, which have similarly suggested that rates of poor outcomes may be underestimated in longer-term follow-ups, as those with more severe strokes die or are lost to follow-up [55, 65]. Although efforts were made to follow-up every individual from the original study still alive at five years, those with more severe cognitive impairments and dementia are likely to be under-represented. The associations between cognitive impairment and outcomes reported here are therefore also likely to be underestimated. Given that only three of the ASPIRE-S stroke survivors who were followed up at five years post-stroke were living in long-term care facilities, nursing home residents are also likely to be under-represented in the ASPIRE-S follow-up study.

We were unable to include help with medication taking in multivariate models due to small numbers and issues with collinearity. The sample size limited the number of potential confounders that could be included in multivariate models and led to a lack of statistical power for detecting statistically significant associations between cognitive impairment and medication adherence. Therefore, this study could not provide a definitive answer on whether or not cognitive impairment is associated with adherence to secondary prevention medications in stroke survivors. Future research should examine associations between cognitive impairment and medication adherence in stroke survivors using a larger sample and longer follow-up period.

Prescription refill data were available for half of our sample. The GMS reimbursement scheme comprises 40% of the population of Ireland; however, owing to the scheme’s eligibility criteria, older adults and those who are socially disadvantaged are over-represented. While over 90% of those aged over 70 are entitled to the scheme, less than 50% of the population under 70 years is eligible [38]. In our sample, 51 of the stroke survivors followed up at five years were under 70 years of age; therefore, medication adherence estimates based on prescription refill data may not be generalizable to younger patients or those from higher socioeconomic groups.

## Conclusion

This study found no statistically significant associations between cognitive impairment and medication adherence post-stroke. Given the difficulties in synthesizing medication adherence research, there is a need to standardize assessment and reporting of medication adherence, ideally using objective methods. Stroke survivors with evidence of cognitive impairment are significantly more likely to receive help with medication taking, which should be taken into account in future studies of medication adherence post-stroke. Involving family members and caregivers may be a legitimate and cost-effective strategy to improve medication adherence in stroke survivors.

## Supporting information

S1 Table Cognitive impairment, medication adherence, and depressive symptoms at five years post-stroke by sex. (DOCX)

S2 Table Demographic and clinical profiles of ASPIRE-S stroke survivors by medication adherence status at five years post-stroke. (DOCX)

S3 Table STROBE checklist. (DOC)
