# Supplementary material for: Cognitive impairment and medication adherence post-stroke: A five-year follow-up of the ASPIRE-S cohort
Source: PLoS One. 2019 Oct 17;14(10):e0223997. doi: 10.1371/journal.pone.0223997 (PMC6797135; doi:10.1371/journal.pone.0223997)
Supplement: S1 Table — (DOCX) [file pone.0223997.s001.docx]

**S1 Table.** Cognitive impairment, medication adherence, and depressive symptoms at five years post-stroke by sex

|  | | **Male** | **Female** | ***p*** |
| --- | --- | --- | --- | --- |
| Cognitive impairment/ aphasia | NINDS | 22 (31.9) | 14 (42.4) | .297 |
|  | MoCA<24 | 31 (44.3) | 15 (48.4) | .703 |
|  | Probable aphasia | 6 (8.6) | 4 (11.8) | .604 |
| Medication non-adherence | Lipids | 4 (11.8) | 4 (21.1) | .365 |
|  | Antithrombotics | 10 (29.4) | 6 (31.6) | .869 |
|  | Antihypertensives | 6 (17.7) | 6 (31.9) | .245 |
|  | Self-report | 13 (19.7) | 9 (31.0) | .228 |
| Depressive symptoms | | 20 (29.4) | 8 (25.8) | .712 |
