# Supplementary material for: Cognitive impairment and medication adherence post-stroke: A five-year follow-up of the ASPIRE-S cohort
Source: PLoS One. 2019 Oct 17;14(10):e0223997. doi: 10.1371/journal.pone.0223997 (PMC6797135; doi:10.1371/journal.pone.0223997)
Supplement: S2 Table — (DOCX) [file pone.0223997.s002.docx]

##### **S2 Table.** Demographic and clinical profile of ASPIRE-S stroke survivors at 5 years by medication adherence status at 5 years

| **Demographics, stroke characteristics and clinical risk factors at six months** | | **Non-adherence at 5 years** | | | | | | | |
| --- | --- | --- | --- | --- | --- | --- | --- | --- | --- |
|  |  | **Antihypertensives (refills)** | | **Antithrombotics (refills)** | | **Lipid modifiers (refills)** | | **Self-report (MARS)** | |
|  |  | **N (%)** | ***p*** | **N (%)** | ***p*** | **N (%)** | ***p*** | **N (%)** | ***p*** |
| **Demographics** | Age (Mean, SD) | 70.3 (9.8) | .644 | 72.2 (10.7) | .765 | 66.9 (9.6) | .172 | 66.7 (11.5) | .017* |
|  | Male | 6 (50.0) | .245 | 10 (62.5) | .869 | 4 (50.0) | .365 | 35 (68.6) | .847 |
|  | Married (vs. not married) | 3 (33.3) | .189 | 7 (50.0) | .786 | 3 (37.5) | .335 | 33 (64.7) | .736 |
| **TOAST classification** | Large artery artherosclerosis | 4 (33.3) | .579 | 2 (12.5) | .229 | 1 (12.5) | .178 | 7 (13.7) | .479 |
|  | Cardioembolism | 3 (25.0) |  | 4 (25.0) |  | 2 (25.0) |  | 14 (27.5) |  |
|  | Small vessel occlusion | 2 (16.7) |  | 4 (25.0) |  | 3 (37.5) |  | 9 (17.7) |  |
|  | Other | 3 (25.0) |  | 6 (37.5) |  | 2 (25.0) |  | 21 (41.2) |  |
| **Bamford classification** | Total anterior circulation stroke | 0 | .405 | 0 | .491 | 0 | .584 | 2 (3.9) | .422 |
|  | Partial anterior circulation stroke | 3 (25.0) |  | 6 (37.5) |  | 3 (37.5) |  | 19 (37.3) |  |
|  | Posterior circulation syndrome | 5 (41.7) |  | 6 (37.5) |  | 1 (12.5) |  | 17 (33.3) |  |
|  | Lacunar syndrome | 4 (33.3) |  | 4 (25.0) |  | 4 (50.0) |  | 11 (21.6) |  |
|  | Unclassifiable | 0 |  | 0 |  | 0 |  | 2 (3.9) |  |
| **Stroke severity** | Moderate or severe | 0 | .033* | 2 (12.5) | .246 | 2 (25.0) | .863 | 6 (11.8) | .379 |
| **Vascular risk factors at 5 years** | Hypertension | 6 (50.0) | .245 | 9 (56.3) | .430 | 5 (62.5) | .916 | 35 (68.6) | .778 |
|  | Elevated total cholesterol | 5 (41.7) | .264 | 8 (50.0) | .025* | 5 (62.5) | .022* | 14 (28.0) | .937 |
|  | Impaired fasting glucose | 4 (33.3) | .336 | 6 (37.5) | .100 | 2 (25.0) | .888 | 8 (16.0) | .377 |
|  | Overweight/obese | 8 (66.7) | .336 | 12 (75.0) | .826 | 5 (62.5) | .293 | 36 (72.0) | .468 |
|  | Smoker | 3 (25.0) | .537 | 4 (25.0) | .453 | 3 (37.5) | .144 | 9 (17.7) | .389 |
|  | History of alcohol abuse | 5 (41.7) | .633 | 7 (43.8) | .430 | 5 (62.5) | .088 | 13 (25.5) | .562 |
|  | History of heart disease | 3 (25.0) | .144 | 6 (37.5) | .569 | 2 (25.0) | .255 | 14 (27.5) | .166 |
|  | History of carotid stenosis | 3 (25.0) | .680 | 4 (25.0) | .616 | 4 (50.0) | .027 | 7 (13.7) | .254 |
|  | Previous/Recurrent Stroke/TIA | 3 (25.0) | .656 | 5 (31.3) | .912 | 3 (37.5) | .625 | 15 (29.4) | .631 |
|  | History of atrial fibrillation | 4 (33.3) | .344 | 6 (37.5) | .454 | 2 (25.0) | .211 | 20 (39.2) | .539 |
|  | Essen Stroke Risk Score (M, SD) | 2.7 (1.0) | .837 | 2.9 (1.4) | .614 | 2.4 (1.3) | .401 | 2.4 (1.3) | .639 |
| **Depression** | Depressive symptoms | 4 (44.4) | .633 | 5 (38.5) | .933 | 2 (25.0) | .424 | 17 (33.3) | .160 |

**p*<.05. TIA: Transient Ischaemic Attack
